# Addressing social resistance in emerging security technologies

**DOI:** 10.3389/fnhum.2013.00483

**Published:** 2013-08-20

**Authors:** Timothy Mitchener-Nissen

**Affiliations:** Department of Security and Crime Science, University College LondonLondon, UK

**Keywords:** technology, social, resistance, ethics, security

## Abstract

In their efforts to enhance the safety and security of citizens, governments and law enforcement agencies look to scientists and engineers to produce modern methods for preventing, detecting, and prosecuting criminal activities. Whole body scanners, lie detection technologies, biometrics, etc., are all being developed for incorporation into the criminal justice apparatus.^1^ Yet despite their purported security benefits these technologies often evoke social resistance. Concerns over privacy, ethics, and function-creep appear repeatedly in analyses of these technologies. It is argued here that scientists and engineers continue to pay insufficient attention to this resistance; acknowledging the presence of these social concerns yet failing to meaningfully address them. In so doing they place at risk the very technologies and techniques they are seeking to develop, for socially controversial security technologies face restrictions and in some cases outright banning. By identifying sources of potential social resistance early in the research and design process, scientists can both engage with the public in meaningful debate and modify their security technologies before deployment so as to minimize social resistance and enhance uptake.

## Introduction

Social constructionism is a sociological theory of knowledge which holds that our knowledge of the world is not derived from observing nature, rather that it is constructed through the social interactions and processes of people (Burr, 2003). By adopting a social constructionist perspective one can comprehend the phenomena of criminality and criminal behavior as existing from the moment individuals and societies began socially constructing and adopting laws which proscribed certain acts or omissions as constituting criminal activities (Newburn, 2007). Under this formulation that which is considered *criminal* can differ spatially (different countries, states, districts, towns have different laws), by ascribed categories (i.e., different laws for different religious groups, genders, sexual orientations, professions and/or social classes) and temporally (laws are not “set-in-stone,” rather are subject to change). Yet while laws can change to reflect both prevailing social views and the organization of activities within a society, the slow pace of this change often results in the law struggling to catch up. The advent of the digital age, the pace of technological development, and the widespread adoption of technologies in many societies all pose challenges for the application of existing laws and the timely creation of new ones.

This paper begins by examining the phenomenon whereby states embrace technologies as solutions or *fixes* for the problem of crime. The negative consequences of this policy in the form of social resistance are then discussed. Finally the question is asked as to why the design and implementation of emerging security technologies continues to repeat mistakes observed in previous technologies? Four answers are provided here, including; (i) the paucity of social education within science, technology, engineering, and mathematics (STEM) courses, (ii) the lack of priority afforded social and ethical issues within the research and design of security technologies, (iii) a general failure by STEM practitioners in comprehending the importance of social acceptability to the technologies they create, and (iv) restricted public engagement.

## Security technologies as technological fixes

The development and interpretation of new technological advancements have been adopted with considerable enthusiasm by governments, law enforcements agencies, universities and private companies as potential methods for preventing, detecting, and prosecuting criminal activities. In this regard they represent *technological fixes* for the social problem of crime; a technological fix is broadly defined as a technological solution for *solving* social problems (Weinberg, 1967) reflecting the views of technological optimists. Technology is presented as a panacea for social problems by being cheaper and more effective than alternative human-centric approaches for dealing with issues which negatively impact society.

The current range of technological fixes designed specifically for addressing crime (hereafter referred to as *security technologies*^2^) continues to increase as scientists and engineers seek to apply the knowledge and approaches of their specific fields to this particular goal. Whole body scanners at airports utilize X-ray backscattering or millimeter wave technology so as to identify metallic and non-metallic objects, plastic and liquid explosives, flora, fauna, drugs, and cash, concealed within or beneath the clothing of passengers (European Commission, 2010; Mitchener-Nissen et al., 2012). Data mining, being the application of database technology and techniques (such as modeling and statistical analysis) to data to identify valid, novel, implicit and potentially useful information and patterns within that data, is employed with the aim of analysing intelligence and detecting terrorist activities, fraud, and other criminal patterns (Tien, 2004; Steinbock, 2005; Schermer, 2011). The use of biometrics enables crime-scene technologies that can assist in the identification and prosecution of offenders (such as DNA databases and fingerprinting technologies), tackling identity fraud, and counteracting illegal immigration (Grijpink, 2006; Goldstein et al., 2008). And to assist in the investigation and prosecution of criminal acts, lie detection technologies designed to directly access brain function (including fMRI and EEG) are trying to be developed by researchers and private companies (Wolpe et al., 2010). This selection represents a tiny snapshot of the cornucopia of security technologies both under development and already implemented.

## Resulting societal resistance

Without further examination it would be tempting to conclude that security technologies do indeed constitute justifications for Weinberg's vision of technological fixes as the solution to social problems. However, the notion of the technological fix has been subject to robust criticism. It has been described as “a quick cheap fix using inappropriate technology that creates more problems than it solves” (Rosner, 2004). The truth of this statement is evident within the social controversies (or in the case of the lie detection technologies, the possible future social controversies) produced by each of the security technology examples provided above. Whole body scanners have been accused of conducting digital strip-searches (Klitou, 2008), and the backscatter variation is to be removed from US airports because of the images produced. Data mining has been associated with both a fear of totalitarian-style state observation, as well as the targeting of individuals by governments (Steinbock, 2005). Different biometric technologies can discriminate against various groups within society and are plagued by the problem of false positives (Hunter, 2005; Whitley and Hosein, 2010). Additionally the UKs DNA database (the largest in the world) has created controversy by holding the details of innocent people and a disproportionate number of samples from ethnic minorities. And the new generation of potential lie-detection technologies have faced criticism over the potential ethical, social, and legal implications of their operation to existing social and legal institutions should they ever be made to definitively and consistently “work.” This social resistance to a security technology begins individually, as solitary citizens question the rationale and/or operation of a particular measure. These may be individuals who actively critique government security policy, those who prioritize privacy and liberty, or as is often the case these are individuals who find themselves adversely impacted upon by a security technology without just cause. For example; individuals who are incorrectly prevented from flying because either they have the same name as another person on a no-fly list, or their details have been added in error to such a list without them being previously notified or provided a way to rectify this error. Recognition of an individual's issues with a security technology can now begin to coalesce into social resistance once knowledge of their plight becomes known to others. The media, lawyers, NGO's, social activists, political figures, and independent commissioners amongst others can all assist is raising awareness here, which in turn can influence other citizens thereby snowballing the effect and reducing support for the security technology in question.

The manifestation of social resistance present in the technologies discussed above represents only a snap-shot of the controversies produced by security technologies which have in the past undermined their social acceptability and widespread uptake. In an on-going examination of security technologies which have evoked social resistance, I have identified numerous recurring controversies which continue to arise within new security technologies with depressing regularity. These can be organized into eight high-level categories; the causing of physical and mental harm, questions of legality, financial costs, liberties and human rights issues, broader public responses, issues of functionality, security and safety issues, and abuse/misuse issues. A selection of commonly recurring controversies includes; privacy concerns, function creep, false positive/negative rates, lack of public trust, the failure of a technology to achieve what its designers claim it can do, and the potential for the technology to be abused by the state.

## Why new security technologies repeat the mistakes of the past

The question which needs addressing here is why have lessons not been learnt such that new security technologies consistently evoke such ethical and social controversy? I suggest there are four complementary elements underpinning the answer to this question. The first is the paucity of social and ethical education within university STEM courses. Within university engineering courses in the UK it is highly likely that a student can (and will) complete their education without ever undertaking a single lecture on the importance of identifying and incorporating social and ethics factors into their work. This is despite the creation of the field of engineering ethics which arose in the early 1980s following a number of technological developments, designs and failures which negatively impacted human wellbeing (Johnson and Wetmore, 2008). The situation is repeated within the hard sciences with the possible exception of medical ethics. For those who counter with the claim that ethics and ethical research is ensured by the presence of university ethics boards; while a particular research or design project may meet all official conduct requirements such that it is considered ethical, this does not mean that what is being undertaken or created will be accepted by the public. The diverse groups which comprise a society ultimately determine what is considered socially or ethically acceptable, and yet university engineering and hard science courses regularly fail new researchers and designers by not equipping them with an understanding of this fact nor the tools to adequately interact with the public.

The second element in the lack of priority afforded social and ethical issues within research and design projects. Interviews with engineers and scientists engaged in the process of designing and developing new security technologies have highlighted a clear hierarchical structure to the design process. For commercial projects it begins with cost; if it is determined that there is not a viable market for a product then it will not be produced. If this test is passed and the project is considered feasible than design specifications are produced in accordance with the client's requirements and the product is created. Similarly with university research projects, the presence of funding and/or the potential for future commercial exploitation dictates the research undertaken. When this is directed toward addressing perceived security deficiencies the focus is on attaining a specific security goal. These processes leave little space for the consideration and incorporation of social and ethical issues—the focus is on “can we achieve what we have set out to achieve,” and not “is this a socially acceptable way of achieving the desired goals” or “are these goals socially acceptable *per se*.”

The third element is a general failure by scientist and engineers to comprehend just how important social acceptability is to the life cycles of their technologies. In the majority, scientists and engineers do not develop an appreciation of the importance of identifying and addressing social concerns until they are confronted by social resistance; a point often reached *after* a product has been released to market.

The fourth element is the challenge of, and the resistance to, achieving effective public engagement in relation to the design of security technologies. The arguments in favor public engagement hold that just as democracy derives its legitimacy through participation, so too will increasing participation within the development of new or controversial technologies help to infuse the finished products with similar legitimacy and reduce societal resistance. The primary argument against is that lay people are handicapped by a lack the technological literacy, or access to and understanding of, security-sensitive intelligence, which together constrain their ability to provide relevant input or make informed decisions. But as Kleinman (2005) highlights, the flawed nature of such views is driven home by the fact that experts^3^ are never value-neutral, unbiased, all-seeing individuals; rather are bounded by the nature of their expert knowledge and will necessarily view a phenomenon from a partial perspective. In other words, experts are handicapped to view the world through blinkers and in this respect have similarities with the very lay public whose input they would seek to exclude.

By introducing socially unacceptable technologies in the first place, trust in both the developers and the end-users (i.e., governments and agencies of the state) is threatened, research and design capacity is diverted from acceptable technologies, and money is wasted that could otherwise have been used for legitimate programmes. The challenge becomes identifying what is acceptable and unacceptable *before* a technology is developed and deployed. By accepting that judgments over acceptability of a technology differ between social groups and that rejection of a technology can lead to its permanent inferiority through neglect (MacKenzie and Wajcman, 1999), the consideration of wider social and ethical issues upstream in the design process to anticipate and mitigate negative social reactions becomes both a valid and logical response.

## Conclusion

The list of technologies developed which have been banned or their use restricted in various societies, (not necessarily because of deficiencies in the underlying science) but because the developers did not seek to anticipate and mitigate social resistance through upstream design modifications is long and growing. It includes backscatter body scanners, instances of data mining, less lethal weapons, polygraph lie detectors, CCTV, national ID card, etc.

To avoid the ignominy of this situation for emerging security technologies developers must take meaningful steps to identify sources of potential social resistance early in the research and design process. This requires truly reflexive engagement with the public to identify concerns which then can be translated into upstream design requirements; thereby heading off social resistance before it coalesces and becomes synonymous with the technology being developed. The enormity of this challenge cannot be overestimated for if a proposed technology cannot by created in such a fashion which respects and reflects the values held within a society, then those developing the technology are wasting valuable time, money and resources on research which will ultimately be rejected.

### Conflict of interest statement

The author declares that the research was conducted in the absence of any commercial or financial relationships that could be construed as a potential conflict of interest.

## Footnotes

^2^By *security technologies* I am referring to the product of an engineering endeavour which seeks to deter, prevent, detect or prosecute crimes, and/or enhance the security of individuals, their property, or the state (including its infrastructure).

^3^In this case STEM practitioners, state officials, and law-enforcement/intelligence officers.
